# Histopathological and immunological spectrum in response evaluation of talimogene laherparepvec treatment and correlation with durable response in patients with cutaneous melanoma

**DOI:** 10.1097/CMR.0000000000000824

**Published:** 2022-04-21

**Authors:** Evalyn E.A.P. Mulder, Jeffrey Damman, Daniëlle Verver, Astrid A.M. van der Veldt, Sam Tas, Tamana Khemai-Mehraban, Kim C. Heezen, Roxane A. Wouters, Cornelis Verhoef, Georges M.G.M. Verjans, Anton W. Langerak, Dirk J. Grünhagen, Antien L. Mooyaart

**Affiliations:** Departments of a Surgical Oncology; b Medical Oncology; c Pathology; d Radiology & Nuclear Medicine; e Viroscience, Erasmus MC Cancer Institute; f Department of Immunology, Laboratory Medical Immunology, Erasmus MC, University Medical Center, Rotterdam, The Netherlands

**Keywords:** granulomatous vasculopathy, melanoma, plasma cells, response, talimogene laherparepvec

## Abstract

Talimogene laherparepvec (T-VEC) is an intralesional oncolytic virotherapy for patients with irresectable stage III–IVM1a cutaneous melanoma. Although this treatment is considered to mainly act through T cell-mediated mechanisms, prominent numbers of plasma cells after T-VEC treatment have been described. The aim was to investigate how often these plasma cells were present, whether they were relevant in the response to treatment, and if these or other histopathological features were associated with durable response to treatment. Histopathological (granulomas, perineural inflammation, etc.) and immunological features [e.g. B cells/plasma cells (CD20/CD138) and T cells (CD3,CD4,CD8)] were scored and correlated with durable tumor response [i.e. complete response (CR) persisting beyond 6 months after treatment]. Plasmacellular infiltrate was examined with next-generation sequencing and immunohistochemistry (IgG, IgM, IgA, and IgD). Plasma cells were present in all T-VEC injected biopsies from 25 patients with melanoma taken at 3–5 months after starting treatment. In patients with a durable response (*n* = 12), angiocentric features and granulomas were more frequently identified compared with patients without a (durable) response (*n* = 13); 75% versus 29% for angiocentric features (*P* = 0.015) and 58% versus 15% for granulomas (*P* = 0.041). There was a class switch of IgM to IgG with skewing to certain dominant Ig heavy chain clonotypes. An angiocentric granulomatous pattern in T-VEC injected melanoma lesions was associated with a durable CR (>6 months). Plasma cells are probably a relevant feature in the mechanism of response but were not associated with durable response.

## Introduction

Talimogene laherparepvec (T-VEC) is an oncolytic virogenetically modified herpes simplex virus type 1 (HSV-1) that has been approved for intralesional treatment of patients with stage IIIB–IVM1a melanoma [1,2]. In the OPTiM phase III randomized trial, the first trial reporting the therapeutic benefit of T-VEC, the overall response rate (ORR) was 26% [3]. Since the introduction in clinical practice, response rates after T-VEC treatment have gradually improved [3,4], most likely due to better patient selection. Especially, a lower tumor burden was associated with a greater likelihood of response [5,6]. Patients can be treated with T-VEC monotherapy up to 1 year, with a median treatment duration of 6 months. Tumor response evaluation of T-VEC injected (sub)cutaneous lesions is usually performed every 3–6 months by punch biopsies. It is a promising treatment as it has only mild and self-limiting side effects [3,7], in contrast to systemic treatments (e.g. immune checkpoint inhibitors and targeted therapies) [7–9].

The modifications in T-VEC, when compared with the virulent HSV-1, consisted of deleting the genes encoding infected cell protein (ICP) 34.5 (neurovirulence factor) [10] and ICP47 (immunogenicity) [11] while inserting the granulocyte-macrophage colony-stimulating factor (GM-CSF) gene [12,13]. These modifications result respectively in direct tumor cell lysis after nonneurovirulent viral replication within cancer cells, enhanced immunogenicity due to increased presentation of viral protein in the major histocompatibility complex class I molecule, and improved response of the innate immune system elicited by GM-CSF [10,14]. In short, the mechanism of T-VEC mechanism is thought to be mainly mediated by T cells and partly by enhancing the innate immune system by GM-CSF [11,15,16]. Interestingly, Richtig *et al*. [17] describe a prominent plasmacellular infiltration in patients (*n* = 3) treated with T-VEC, which is not to be expected from the working mechanism. Also, a pseudolymphomatous reaction, an inflammatory response with a relatively large number of B cells, has been reported (*n* = 1) [18]. Everett *et al*. [19] also described the presence of plasma cells in the first histopathological case series of patients treated with T-VEC (*n* = 5), but mainly focused on the presence of granulomas, as did Lee *et al*. [20] (*n* = 3). Therefore, it is unclear how often this plasmacellular/B cell/humoral response is present and so far analysis of a large series of the histopathological spectrum of T-VEC is lacking. The presence of these humoral responses (including B cells/plasma cells) is interesting as they have been identified as an indicator of persistent response to other forms of immunotherapies, such as immune checkpoint inhibitors [21,22]. As these checkpoint inhibition immunotherapies are also mainly T cell-mediated, this may also be an indicator for persistent response in T-VEC treated patients.

Therefore, we investigated how often a humoral response pattern is seen and when present, whether this reflects a specific response to the tumor. Furthermore, we aimed to determine whether this humoral response or another histopathological/immunological patterns are associated with (durable) response to T-VEC treatment.

## Materials and methods

### Patient selection and clinical features

T-VEC treatment was introduced in July 2017 at the Erasmus MC Cancer Institute. To evaluate pathological and clinical responses to T-VEC treatment, tissue and clinical data from patients with melanoma who started treatment between July 2017 and August 2019 at the Erasmus MC Cancer Institute were retrieved. Patients without one or more biopsies from a T-VEC injected melanoma lesion were excluded. Data on patient characteristics (age and sex), treatment (duration, response, and side effects), and follow-up (recurrence and survival) were retrieved from the medical records, whereas details on the primary melanoma (e.g. Breslow thickness and ulceration) were obtained from the patients’ pathology reports.

In these patients, intralesional T-VEC injection had been performed with 10^6^ plaque-forming units (PFU)/ml, followed 3 weeks later by biweekly T-VEC 10^8^ PFU/ml injections, up to 1 year, with a maximum of 4 ml per treatment (depending on the number and size of lesions). For our research question, tissue from at least one T-VEC injected (sub)cutaneous lesion had to be available per patient. From these patients, clinical outcomes to T-VEC were divided into complete response (CR) for over 6 months versus no (complete) response, including progressive disease (PD), stable disease (SD), partial response (PR), and CR ≤ 6 months. Best ORR was defined as the number of patients with CR or PR. Follow-up status was assessed and patients were categorized as alive without evidence of disease (either through T-VEC alone or other type(s) of treatment), alive with disease, or died of disease (here: melanoma) or other causes.

### Evaluation of histopathological features

Hematoxylin and eosin (H&E) staining of formalin-fixed paraffin-embedded (FFPE) sections (3 μm) were performed for routine diagnostics. For additional staining and evaluation of biopsies from T-VEC tissue, sections were collected from the archives of the pathology department of the Erasmus MC Cancer Institute. If available, pretreatment biopsies were also obtained. Histopathologic features were scored by two dermatopathologists without prior knowledge of clinical outcomes. Relevant histological features were scored as follows: amount of infiltrate [low (<10%), moderate (10–50%), and high (>50%)]; degree of infiltrate [superficial (1), deep (2), or both (3)]; and the presence of neutrophil granulocytes/eosinophilic granulocytes/extravasation of erythrocytes/melanophages [not/barely (0), moderate (1), and many (2)]. Presence or absence of tumor cells, granulomas, tertiary lymphoid structures (TLS; clustering of B cell follicles surrounded by T cells within nonlymphoid tissue), perivascular, interstitial, perineural, and angiocentric inflammations were scored dichotomously (no/yes), as well as a response adjacent to sweat glands, hair follicles and sebaceous glands, viral changes of the epidermis, and other changes of the epidermis. Disagreements were resolved by discussion until both pathologists reached a consensus. The angiocentric character of the inflammatory lesion was defined as an infiltrate with a concentric pattern around the vessel, which could be histiocytic, a pattern also referred to as granulomatous vasculitis [23], but also plasmacellular.

### Immunohistochemistry of the immune infiltrate

For routine diagnostics, SOX10 or Melan-A or both were used to identify (metastatic) melanoma cells. The corresponding FFPE tissues were retrieved to which prep kits antibodies were applied in the BenchMark ULTRA IHC/ISH System (Roche Tissue Diagnostics, Ventana, Tucson, Arizona, USA). To distinguish between an HSV-1-like reaction versus a response that is more similar to immunotherapy (including TLS formation), we identified CD3 (T cell receptor–associated molecule; pan T cells), CD4 (T helper cells), and CD8 (cytotoxic T cells) versus CD20 (B cells), and CD138 (plasma cells), respectively. The following mAb clones (moAb, all from Ventana) were used: 2GV6 (αCD3), SP35 (αCD4), SP57 (αCD8), and L26 (αCD20). For CD138, the EP201 moAb (Cell Marque) was used. In all biopsies showing cells morphologically consistent with plasma cells, the following moAbs were applied: anti-IgG (Cell Marque, 0828), and anti-IgM, anti-IgA, and anti-IgD (DAKO 760-2652, 760-2654, and 760-2654, respectively).

The magnitude of CD3 infiltrate was scored ordinally: <25% (1), 25–75% (2), and >75% (3) of the infiltrate. The density of CD20 and CD138 positive cells were given per mm^2^ in high-power field. CD20 was scored ordinal: <10 (0), 10–20 (1), and >20/clustered (2). Also, CD138 was scored ordinally: <10 (0), 10–40 (1), and >40 (2). Furthermore, the relative proportion from 0 to 1 between CD3/CD20, CD3/CD138, and CD4/CD8 was estimated. By multiplying the semiquantitative total degree of infiltration (as assessed on H&E, i.e. 1, 2, or 3) with these relative proportions (i.e. 0–1), the relative degree of infiltrate for these specific subpopulations (of immune cells) was estimated. This calculated score ranges from 0 to 3, with 0 representing the least amount of infiltration and 3 the highest.

#### Herpes simplex virus type 1-specific PCR and immunohistochemistry

First, HSV-1 immunohistochemical staining was performed using two different antibodies, an anti-HSV-1 ICP8 moAb (clone 10A3, Sigma-Aldrich) and an anti-HSV-1 ICP 8 poAb (Agilent), on a subset of samples showing histopathological granulomas and perineural inflammation, as these features are also described in (late) herpetic reactions. Second, on these same samples, to identify the presence of HSV-1 DNA in these lesions, DNA was extracted from FFPE tissues, to determine which HSV type was present. In addition, cycle threshold (Ct) values were calculated (high Ct values represent a low viral load and vice versa).

#### Plasma cell immunohistochemistry and clonality analysis

To gain more insight into the local B cell response in T-VEC treated tumors, we identified patients with multiple biopsies at a given time point with a clear presence of plasma cell formation at a given time point. In these biopsies, in addition to the absence/presence of immunoglobulins (Ig) (e.g. IgG, IgM, IgA, and IgD) heavy and light chains (kappa/lambda) were assessed. Furthermore, next-generation sequencing (NGS)-based Ig clonality analysis was performed to further classify these plasma cells with respect to their clonality: poly-, oligo- or monoclonal plasma cell infiltrate [24]. DNA was isolated from multiple FFPE biopsies from three different patients (A, B, and C). To reduce amplification efficiency due to blocking substances, DNA was 1:10 diluted prior to amplification with Ig-specific primers for both complete heavy chain variable region-joining region of heavy chain (FR3) and incomplete heavy chain diversity region-joining region of heavy chain (DH-JH) rearrangements. The latter target was added on purpose based on the concept that the FR3 region of complete Ig heavy chain (IGH) rearrangements in plasma cells is often containing many somatic hypermutations, whereas DH-JH rearrangements do not suffer from a high somatic hypermutation load. A detailed description of library preparation, high-throughput sequencing, and data analysis for NGS-based Ig clonality detection was described earlier [24]. In short, following library preparation, sequencing was performed on the IonTorrent S5 platform, and data analysis was done using the ARResT/Interrogate immunoprofiler tool [25].

### Statistics

All statistical analyses were performed using SPSS version 26.0 (IBM, Armonk, New York, USA). For clinical response, patients were categorized as having no (lasting) response versus having a CR for at least 6 months. Subgroup analyses were performed for comparing histological features of biopsies before and after T-VEC treatment initiation. Differences between groups were calculated using χ^2^ tests, Fisher’s exact tests, or nonparametric Mann–Whitney U tests, as appropriate. Where data were missing or unknown, an ‘unknown’ subcategory was created. A *P*-value <0.05 (two-sided) indicated statistical significance.

## Results

Between July 2017 and August 2019, T-VEC treatment had been initiated in 30 patients with advanced melanoma. Sufficient biopsy material was available from 25 patients. The median age was 72 years [interquartile range (IQR), 65–79], and the majority presented with irresectable stage IIIC melanoma (13 out of 25; 52%). Table 1 provides an overview of patients recruited and their tumor characteristics. The median time to first biopsy for pathological evaluation of T-VEC effects was 5 months (IQR, 3–5 months) and the majority of T-VEC injected biopsies were free from tumor tissue (23 of 25; 92%). Best ORR to T-VEC treatment was 88.4%, with a CR in 61.5%. In nearly half of the patients (12 of 25; 48%), CR lasted more than 6 months versus 52% without a (lasting) response (PD, SD, CR ≤ 6 months; *n* = 13). At baseline, patient and tumor characteristics were not very different between patients with and without a (durable) response.

**Table 1:** Description of clinical characteristics

Patient and tumor characteristics	Included patients (*n* = 25)	CR > 6 months	PD/SD/PR/CR ≤ 6 months	*P*-values (of interest)
		*n* = 12 (48)	*n* = 13 (52)	
Age in years	72 (65–79)	72 (64–86)	72 (66–77)	0.723
Sex, female	14 (56)	5 (42)	9 (70)	0.238
Substage (AJCC, 8th edition)				
IIIA	8 (32)	5 (42)	3 (23)	0.220
IIIB	13 (52)	6 (50)	7 (54)	
IIIC	3 (12)	1 (8)	2 (15)	
IIID	0	0	0	
IVM1a	1 (4)	0	1 (8)	
Breslow, mm	*n* = 23	*n* = 11	*n* = 12	0.235
	2.3 (1.2–4.0)	1.7 (1.2–3.0)	3.3 (1.3–4.5)	
Ulceration^a^	*n* = 17	*n* = 9	*n* = 8	0.335
Present	6 (35)	2 (22)	4 (31)	
Absent	11 (65)	7 (78)	4 (31)	
Location metastases				
Extremities	21 (84)	10 (83)	11 (85)	1.000
Head and neck	3 (12)	1 (8)	2 (15)	
Trunk	1 (4)	1 (8)	0	
Number of metastases^b^ (category)				
<20	22 (88)	11 (92)	11 (85)	1.000
>20	3 (12)	1 (8)	2 (15)	
Type of metastases				
Cutaneous only	10 (40)	5 (42)	5 (39)	0.794
Subcutaneous	8 (32)	4 (33)	4 (31)	
Lymph node(s)	7 (28)	3 (25)	4 (31)	
Time to first biopsy, months	5 (3–5)	4 (3–5)	5 (4–6)	0.404
Diameter of largest metastasis, mm	11 (8–23)	11 (7–20)	15 (22–28)	0.891
Pretreatment melanoma metastases^c^				-
Surgical excision, yes	19 (73)	8 (67)	10 (77)	
Radiotherapy, yes	0	0	0	
ILP, yes	6 (23)	0	6 (46)	
Systemic therapy, yes	3 (12)	1 (8)	2 (15)	
Mutation status^d^	*n* = 7	*n* = 1	*n* = 6	-
BRAF-mutant	4	0	4 (67)	
BRAF-wild type	1	1 (100)	0	
NRAS	2	0	2 (33)	
Follow-up status				-
NED	13 (52)	11 (92)	2 (15)	
AWD	8 (32)	0	8 (62)	
DOD	3 (12)	0	3 (23)	
DOC	1 (4)	1 (8)	0	
Follow-up interval after last T-VEC dose, months	18 (9–25)	18 (9–24)	18 (9–27)	0.549

Results are given as numbers (%) or median (IQR).

AJCC, American Joint Committee on Cancer; AWD, alive with disease; CR, complete response; DOC, died of other cause; DOD, died of disease; ILP, isolated limb perfusion; IQR, interquartile range; NED, no evidence of disease; PD, progressive disease, PR, partial response; SD, stable disease; T-VEC, talimogene laherparepvec.

a Primary melanoma of unknown origin (*n* = 1), PA report of primary melanoma not available (*n* = 5), ulceration not mentioned in PA report (*n* = 2).

b Number of satellite/in-transit/lymph node metastases.

c Some patients were treated with different treatment modalities before T-VEC was started.

d Mutation status was determined in a selection of patients.

### Histopathological and immunohistochemical findings

**Fig. 1:**
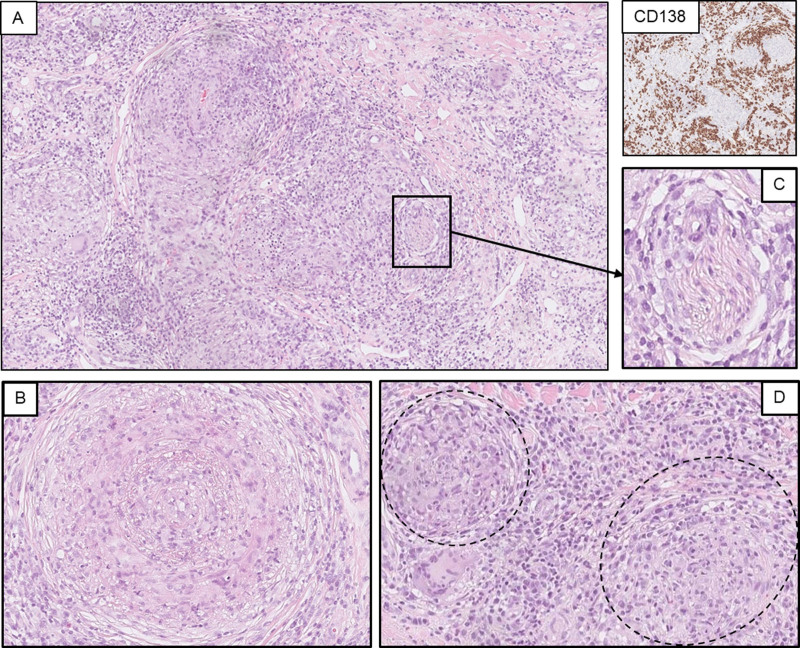
A representative case of successful treatment with T-VEC. This H&E shows a complete response (i.e. no residual melanoma tumor tissue) after 12 months of T-VEC treatment and the presence of (a) diffuse plasma cells [CD138]. Plasma cells can be recognized by their oval shape, round and eccentric nucleus with coarse chromatin, a prominent perinuclear hof, and abundant basophilic cytoplasm. Also, a classical HSV-associated histopathological pattern was seen, including (b) angiocentric and (c) perineural features, and (d) granulomas. H&E, hematoxylin and eosin; T-VEC, talimogene laherparepvec.

**Fig. 2:**
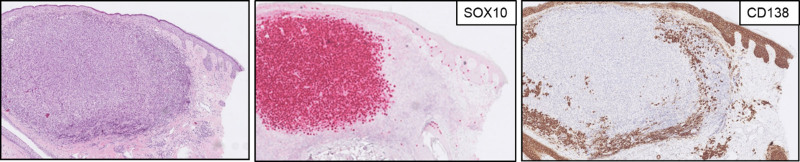
Plasma cell formation after 3 months of T-VEC treatment. H&E shows a subcutaneous in-transit metastasis, identified by (SOX10), with peritumoral plasma cells (CD138). H&E, hematoxylin and eosin; T-VEC, talimogene laherparepvec.

In biopsies from T-VEC injected sites, localization of the infiltrates shifted from solely superficial to deep, occasionally in combination with superficial (Supplementary Fig. S1, Supplemental digital content 1, http://links.lww.com/MR/A295). Infiltration mainly consisted of T cell (associated) immune cells (reflected by the presence of CD3, CD4, and CD8), but a specific pattern within T cells was not associated with durable response. Plasma cells in varying degrees were present in all biopsies. Figure 1 shows a representative case of successful treatment with T-VEC (i.e. no residual melanoma tumor tissue after 12 months of treatment) with the formation of diffuse plasma cells in combination with perineural features, and both angiocentric and ‘classical’ granulomas. A comprehensive overview of histopathological features is provided in Table 2. Formation of a TLS was observed in a subset of patients treated with T-VEC, an example of such a TLS can be seen in Supplementary Fig. S2, Supplemental digital content 1, http://links.lww.com/MR/A295. Interestingly, the relative degree of plasma cells [CD138, 1.0 (0.3–1.4)] was comparable to the relative degree of cytotoxic T cells [CD8, 1.2 (0.75–1.5)]. Figure 2 shows that this plasma cell formation was already observed at 3 months after T-VEC start. However, no relation with a humoral response pattern (e.g. plasma cells, B cells, and TLS) and durable response was seen. In contrast, both angiocentric features and granulomas were more frequently identified in patients with CR (>6 months) compared with patients without a durable response [75% vs. 29% (*P* = 0.015) and 58% vs. 15% (*P* = 0.041), respectively]. Other histopathological findings such as melanophages were equally found in both groups (*P* = 0.633), whereas typical epidermal changes after a natural HSV infection were not observed. A pretreatment biopsy of an in-transit metastasis can be found in Supplementary Fig. S3, Supplemental digital content 1, http://links.lww.com/MR/A295 to demonstrate that this plasma cell infiltrate was not present prior to intralesional T-VEC injections.

**Table 2:** Overview histopathological features

Histopathological features	All patients (*n* = 25)	Clinical outcome		
		CR > 6 months	PD/SD/PR/CR ≤ 6 months	*P*-value
		*n* = 12 (48)	*n* = 13 (52)	
Amount of infiltrate				0.836
Low	4 (16)	1 (8)	3 (23)	
Moderate	10 (40)	6 (50)	4 (31)	
High	11 (44)	5 (42)	6 (46)	
Localization of infiltrate (=degree)	*n* = 24			1.000
Deep (whether or not in combination with superficial)	20 (83)	10 (83)	10 (77)	
Superficial only	4 (17)	2 (17)	2 (15)	
CD3 relative^a^	3.0 (2.0–4.0)	2.5 (2.0–5.5)	3.0 (2.0–4.0)	1.000
CD4 relative^a^	1.0 (0.8–1.5)	1.1 (0.85–1.5)	1.0 (0.70–1.5)	0.847
CD8 relative^a^	1.2 (0.75–1.5)	1.0 (0.75–1.5)	1.2 (0.75–1.5)	0.640
CD20 relative^a^	0.30 (0.15–0.60)	0.25 (0.15–0.60)	0.40 (0.10–0.75)	0.719
CD138 relative^a^	1.0 (0.30–1.4)	0.90 (0.35–1.4)	1.0 (0.30–1.4)	0.970
Interstitial, yes	20 (80)	11 (92)	9 (70)	0.322
Perineural, yes	9 (36)	5 (42)	4 (31)	0.688
Angiocentric, yes	14 (56)	10 (83)	4 (31)	0.015
Granulomas, yes	9 (36)	7 (58)	2 (15)	0.041
Possible TLS, yes	16 (64)	9 (75)	7 (54)	0.411
Perivascular, yes	25 (100)	12 (100)	13 (100)	1.000
Eosinophilic presence				0.082
Not/barely	22 (88)	12 (100)	10 (77)	
Moderate	3 (12)	0	3 (23)	
Many	0	0	0	
Melanophages				0.768
Not/barely	12 (48)	5 (42)	7 (54)	
Moderate	8 (32)	5 (42)	3 (23)	
Many	5 (20)	2 (17)	3 (23)	
Extravasation of erythrocytes				0.563
Not/barely	22 (88)	11 (92)	11 (85)	
Moderate	2 (8)	1 (9)	1 (8)	
Many	1 (4)	0	1 (8)	
Infiltrate next to hair follicles	*n* = 23			0.296
Yes	1 (4)	0	1 (9)	
No	22 (96)	12 (100)	10 (91)	
Infiltrate next to sweat glands	*n* = 23			0.156
Yes	14 (61)	9 (75)	5 (46)	
No	9 (39)	3 (25)	6 (55)	
Infiltrate next to sebaceous glands	*n* = 23			0.296
Yes	1 (4)	0	1 (9)	
No	22 (96)	12 (100)	10 (91)	

Biopsies were performed for histopathological evaluation of T-VEC effects (median time to first biopsy was 5 months).Clinical outcomes were divided into CR for over 6 months versus no (complete) response, including PD, SD, PR, and CR ≤ 6 months.

CR, complete response; PD, progressive disease; PR, partial response; SD, stable disease; TLS, tertiary lymphoid structure; T-VEC, talimogene laherparepvec.

a The relative degree of infiltrate for these specific subpopulations (of immune cells) was estimated, by multiplying the semiquantitative total degree of infiltration (as assessed on H&E, i.e. 1, 2, or 3) with the relative immune cell proportions (i.e. 0–1)

### Herpes simplex virus type 1-specific real-time PCR and immunohistochemistry

To gain a more in-depth understanding of the effect of intralesional injection with an oncolytic virogenetically modified HSV-1 on melanoma cells, additional analyses were performed in T-VEC injected biopsies. On three biopsies that showed granulomas and perineural growth on H&E sections, conventional immunohistochemistry using HSV-specific antibodies was performed, providing no evidence of a sustained lytic HSV-1 infection. This notion is supported by the detection of very low amounts of HSV-1 DNA detected by virus-specific real-time PCR on DNA isolated from the same biopsies, which represent remnants of a cleared HSV-1 infection (data not shown). The absence of a positive HSV-1 IHC signal indicates that the low amounts of HSV-1 DNA detected in the same biopsies represent remnants of a past HSV-1 oncolytic virus injection.

### Plasma cells immunohistochemistry and clonality analysis

**Fig. 3:**
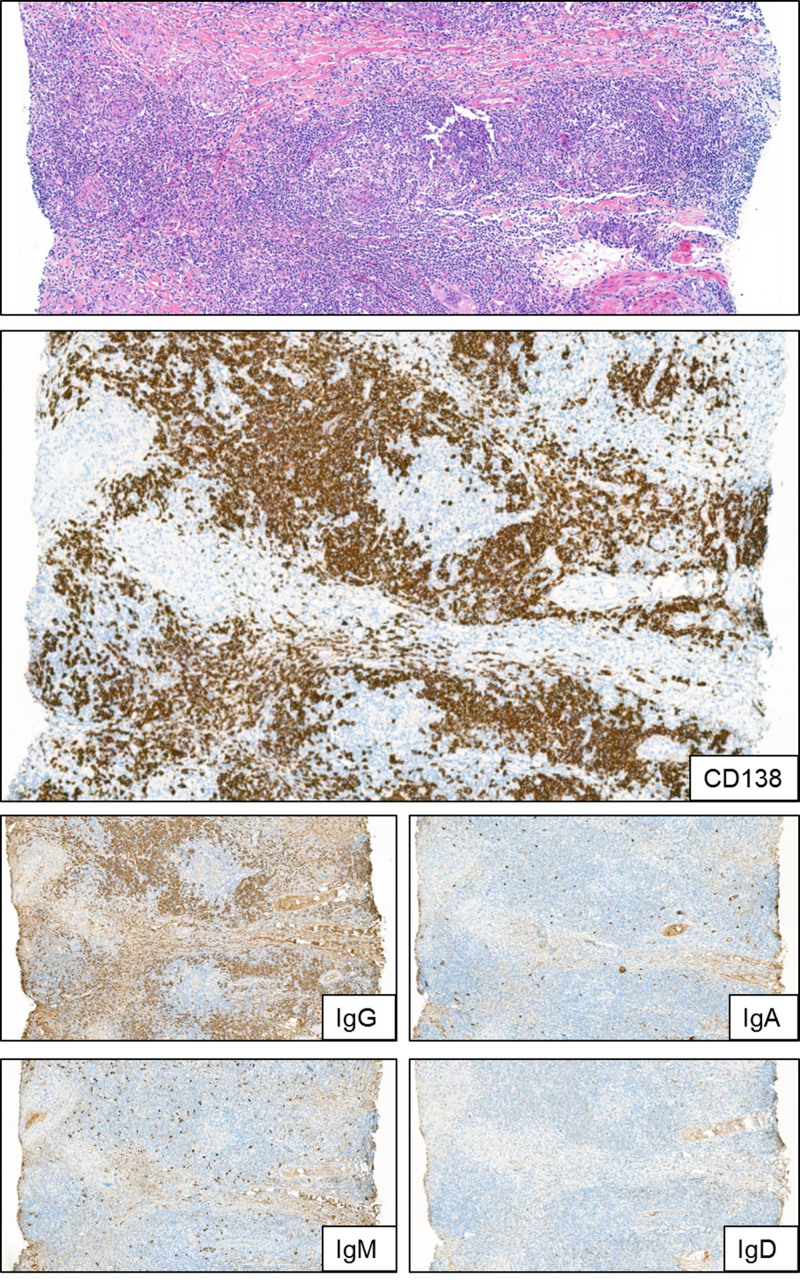
Immunoglobulin subclasses expressed by plasma cells in T-VEC injected biopsies. H&E showing prominent and diffuse plasma cell formation (CD138), predominantly IgG isotype. Other isotypes (IgM, IgA, and IgD) were barely present. H&E, hematoxylin and eosin; Ig, immunoglobulin; T-VEC, talimogene laherparepvec.

In three patients in whom multiple biopsies were performed at a given time point (ranging from 8 to 13 T-VEC treatments), further analysis revealed plasma cell infiltration of the IgG isotype. Immunoglobulin subtypes IgM, IgA, and IgD were not or only incidentally detected (Fig. 3). To further evaluate the clonality of the intratumor plasma cell infiltrates, NGS-based Ig clonality analysis was performed on DNA isolated from multiple biopsies obtained at a single time point from three patients (patients A, B, and C). In two of three patients (A and C), hardly any immunoglobulin heavy chain variable-immunoglobulin heavy joining (IGHV-IGHJ) sequence reads were obtained, which contrasts the readily detectable IGHD-IGHJ reads in all three patients. In two patients (A and B), a poly-/oligoclonal profile with recurrent dominant IGHD-IGHJ or IGHV-IGHJ clonotypes was detected in multiple biopsies within one patient. More detailed comparison of biopsies from multiple sites in these two patients revealed considerable overlap in the dominant IG clonotypes, either IGHD-IGHJ or IGHV-IGHJ, between different biopsy sites in one patient. In patient C, the IGH profile was even more skewed and almost monoclonal, with a dominant IGHD-IGHJ clonotype (63% of reads) and a background of a few minor IGHD-IGHJ clonotypes. In addition, an oligoclonal IGHV-IGHJ profile was also observed with multiple dominant clonotypes. Unfortunately, in this patient, two biopsies from other sites were not informative due to low DNA yield and poor DNA quality, which prohibited further evaluation of the consistency of the profile at different anatomic sites. To rule out a technical artifact due to preferential amplification of few templates in especially the patient with the monoclonal IGHD-IGHJ clonotype, multiple replicates were performed. Replicates always showed the same dominant IGHD-IGHJ clonotype (53–71% in patient C) or the same minor clonotypes (IGHD-IGHJ and IGHV-IGHJ in patients A and B, respectively). Collectively, these data would point to skewing of the Ig repertoire, suggestive of a nonrandom distribution of plasma cells in the biopsies.

## Discussion

In this study, we described detailed immunological and histopathological features in patients with irresectable stage III-IVM1a cutaneous melanoma receiving T-VEC therapy. In addition, we correlated these features with clinical outcome. Most biopsies showed a prominent humoral, mainly plasmacellular, response to intralesional T-VEC treatment, even at an early stage. The apparent class switch of IgM to IgG in plasma cells in combination with skewing to certain dominant IGH clonotypes in T-VEC injected skin biopsies suggests that these plasma cells are not random but potentially contributed to the T-VEC-induced local immune response [26]. This is unexpected as the therapeutic immune response to T-VEC therapy would be expected to primarily involve T cells [27]. Contrary to what has been described in studies on other forms of immunotherapy [21,22,28], we found no correlation between this humoral pattern and a persistent immune response to T-VEC. However, an association was found between durable response and angiocentric (granulomatous) features and granulomas. We confirm that HSV-1 PCR can detect remnants of T-VEC in the tissue, but no HSV-1 protein was detected with immunohistochemistry in the reactive infiltrate or surrounding tissue.

Currently, the role of a humoral response in T-VEC–associated inflammation is poorly understood. After tumor lysis, virus particles and tumor antigens are presented to dendritic cells (DCs). These DCs (presenting MHC class II) with the help of T helper (CD4+) cells induce a B cell response. This would be expected to primarily result in a systemic B cell response in secondary lymphoid organs (a lymph node) and not lead to prominent local accumulation of B cells and plasma cells or TLS formation, usually a local response. Plasma cells usually migrate to the bone marrow after activation, and abundant local response would be unexpected as a result of only tumor lysis. Abundant local plasma cells are described in Lues, Borrelia, and Leishmania infections, but usually are not such a prominent feature in herpes simplex infections. It has also been described that T-VEC can trigger a systemic antitumor immune response by reduced suppression of antigen presentation, leading to an increased presentation of MHC class I [10]. Such an upregulation, however, would result in a cytotoxic immune response [29], rather than an increase of B cells and plasma cells. In our biopsies, this cytotoxic (CD8+) response was present, but to a similar degree as the humoral response pattern. Also, the integrated GM-CSF in T-VEC mainly recruits monocytes and lymphocytes to stimulate the immune system [11,30] and does not provide an explanation for these abundant local plasma cells. As there was no correlation with durable response, the presence of this humoral pattern is likely to have a different role than in other T cell-mediated immunotherapies. Therefore, more research is needed to further understand the reason of this humoral response, as it seems to be relevant to the treatment response, instead of mainly focusing on the DCs and T cells [31].

Granulomas, especially in an angiocentric pattern (also called granulomatous vasculopathy/vasculitis) were associated with durable response to T-VEC treatment. These (angiocentric) granulomas have been described in late response to herpes (mainly herpes zoster [23]). Although the neurovirulent ICP34.5 gene was deleted in T-VEC, this inflammatory pattern might still be related to herpes. Potentially, angiocentric granulomas are a result of a more prominent antiviral response in these patients. We speculate that a possible explanation for a durable response to T-VEC could be a result of an enhanced immune response to T-VEC due to a previous (latent) infection with HSV-1. Although all patients were asked if they ever had a cold sore before starting treatment and the majority did not, it is well known that the majority of HSV-1 infected people is asymptomatic [32]. Further research is thus needed to elucidate this hypothesis.

### Strengths and limitations

This represents the largest series to evaluate detailed T-VEC induced histopathological features of patients with irresectable cutaneous melanoma. Furthermore, to the best of our knowledge, this is the first time histopathological and immunological features were correlated with durable clinical responses in patients treated with T-VEC. We quantified the inflammatory infiltrate and analyzed the plasmacellular component by NGS and investigated the presence of HSV-1 (by both PCR and immunohistochemistry), making this the most detailed series of T-VEC response evaluation biopsies so far. Our response rates to T-VEC were in line with those described previously [4], with a best ORR of 88.4%. Inherent to the research question, tissue from a T-VEC injected (sub)cutaneous lesion had to be available for each patient. In patients treated with T-VEC, both subcutaneous and nodal metastases are negative predictors for outcome [6]. When no biopsies from (sub)cutaneous lesions were available, this could mean that there were only nodal lesions. Furthermore, in the case of rapidly PD (beyond the locoregional lymph node station), taking cutaneous biopsies is of no consequence for the treatment plan. Not including these patients (*n* = 3) will have caused some sort of selection bias with regard to best ORR. However, the main focus was the histopathological and immunological spectrum in response to T-VEC and correlation with durable response in patients with cutaneous melanoma, not the best ORR. In addition, baseline (and longitudinal) sample collection was not available in the majority of patients. Therefore, it was not possible to describe T-VEC–induced changes over time in more detail.

### Future perspectives

In addition to the histopathological evaluation of T-VEC injected biopsies to clarify the T-VEC mechanism, it would be relevant to evaluate the systemic response in more detail (e.g. blood samples and radiological assessments). Moreover, the role of prior HSV-1 infection (and subsequent presence of serum HSV IgG antibodies) is yet to be determined. Through a better understanding of the exact mechanism of action in this anticancer immunotherapy, we could be able to adjust the treatment strategy to ensure that more people respond and have a durable response. In line with this, we aim to identify patients who will not respond to T-VEC monotherapy and might benefit from other forms of (systemic) treatments, whether or not combined with T-VEC treatment. The role of immunotherapy in oncology treatment strategies is evolving and combining immunotherapy with T-VEC seems promising [33–35]. In patients treated with systemic immunotherapy, predictive biomarkers are being explored that may contribute to (durable) antitumor immunity and subsequent response to treatment [36]. We believe that further research to unravel T-VEC’s exact working mechanism is needed to understand its effect (both local and systemic) and ultimately optimize the benefit of treatment in general, not limited to T-VEC.

### Conclusion

Based on what we observed in histopathological evaluation of biopsies from patients with T-VEC injected metastatic melanoma, a humoral response (including B cells, plasma cells, and occasionally a TLS) was present in the vast majority of patients. This pattern was found to have no predictive value in terms of (durable) response, but we found that this is likely a relevant feature in the response mechanism. An (angiocentric) granulomatous pattern was associated with a (durable) response (i.e. CR > 6 months). This pattern might be related to a previous latent HSV-1 infection, but this is still highly speculative. Collectively, these results imply that the exact mechanisms of action of T-VEC immunotherapy are still incompletely disclosed and require further investigation. A better understanding of T-VEC’s working mechanism can help to identify those patients who will respond to T-VEC and those who will not and ultimately can be used to optimize treatment regimens in patients with irresectable cutaneous melanoma.

## Acknowledgements

Ethics approval: this study was performed according to the Declaration of Helsinki and approved by the institutional review board of the Erasmus MC (MEC-2020-0294) and patient data were used according to ‘The Code for Proper Secondary Use of Human Tissue’ and ‘The Code of Conduct for the Use of Data in Health Research’ as stated by the Federation of Dutch Medical Scientific Societies. None of the patients objected to coded use of their residual tissue for scientific research.

### Conflicts of interest

A.V.: consultant and advisory board member BMS, MSD, Merck, Ipsen, Eisai, Pfizer, Novartis, Pierre Fabre, Sanofi, and Roche. All paid to institute and unrelated to current work. A.L.: research support and speaker Roche-Genentech and Janssen; all unrelated to the here presented work. E.M., J.D., D.V., S.T., T.K.-M., K.H., R.W., C.V., G.V., D.G., and A.M. have declared no competing interests.

## Supplementary Material


