# Supplementary material for: Histopathological and immunological spectrum in response evaluation of talimogene laherparepvec treatment and correlation with durable response in patients with cutaneous melanoma
Source: Melanoma Res. 2022 Apr 21;32(4):249–59. doi: 10.1097/CMR.0000000000000824 (PMC9245556; doi:10.1097/CMR.0000000000000824)

Supplementary material

S1. Deep and superficial infiltrate after successful treatment with T-VEC.

This H&E shows a complete response (i.e., no residual melanoma tumor tissue) to T-VEC.

Abbreviation: T-VEC, talimogene laherparepvec.

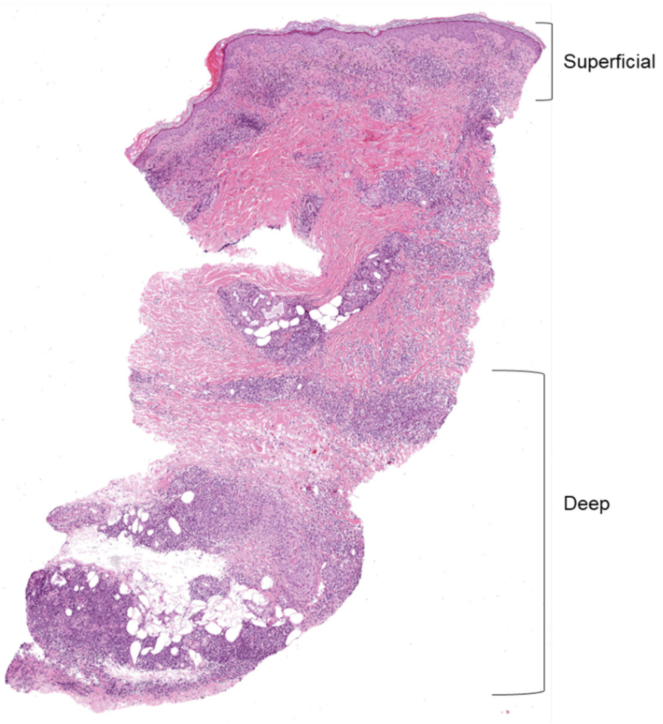

S2. Formation of TLS in biopsies from T-VEC injected lesions.

H&E shows infiltrate clustering of B cell follicles surrounded by T cells within non lymphoid tissue, forming a TLS. Additional immunohistochemistry identified CD20 [B cells] and CD3 [T cells].

Abbreviations: H&E, Hematoxylin and eosin TLS, tertiary lymphoid structure; T-VEC, talimogene laherparepvec.

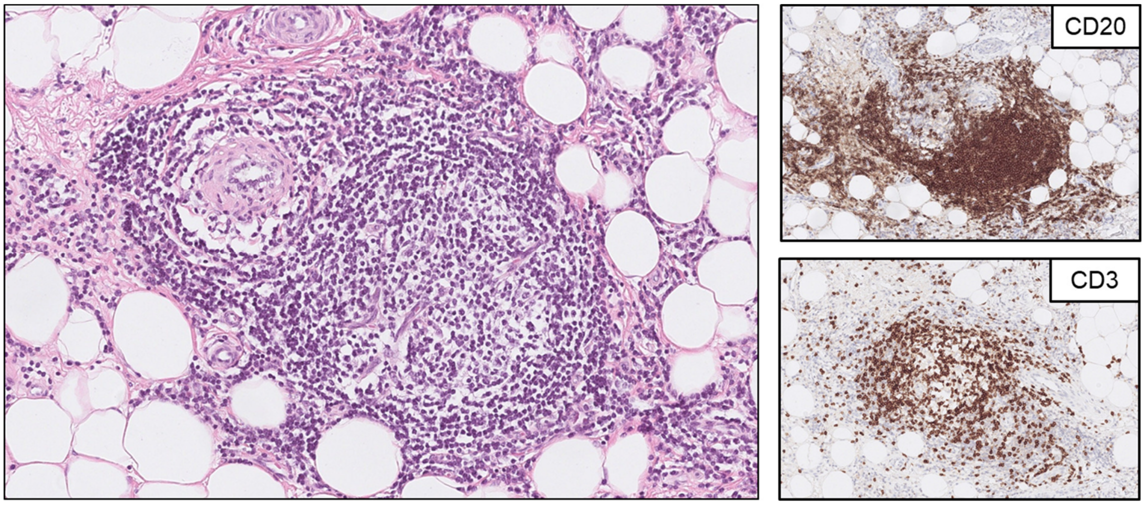

S3. Pre-treatment biopsy of a subcutaneous in-transit metastasis

A, overview; intact epidermis and subcutaneous accumulation of melanocytes with nuclear atypia. B, zoomed in.

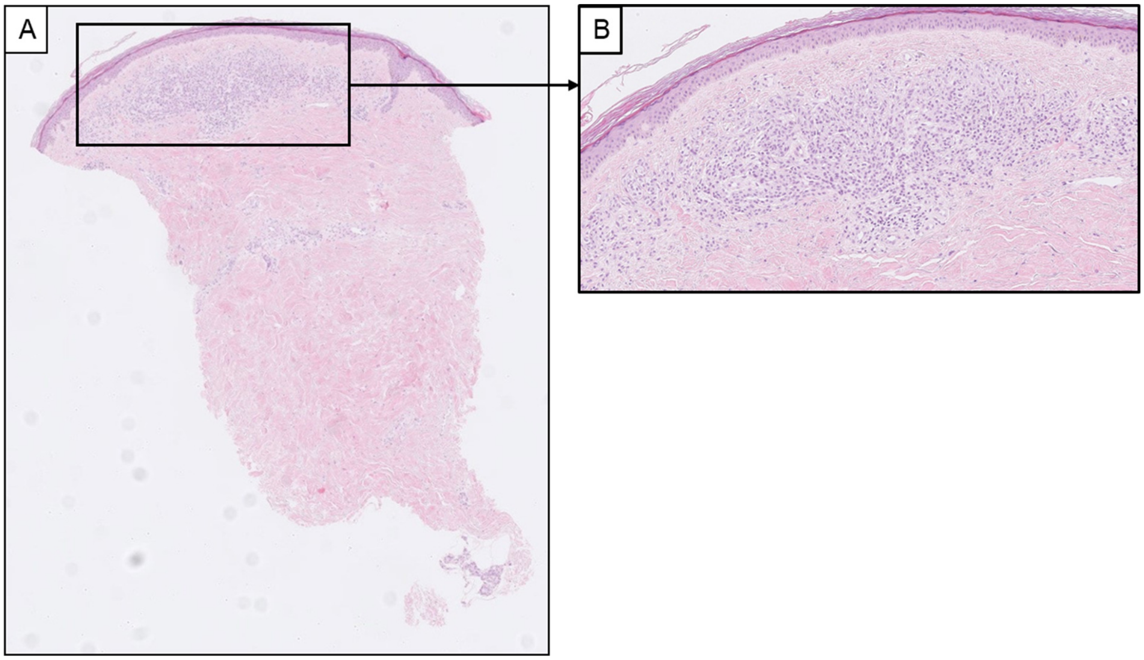

Supplement: Supplementary file 1 [file mr-32-249-s001.pdf]
